# Diagnostic leukapheresis reveals distinct phenotypes of NSCLC circulating tumor cells

**DOI:** 10.1186/s12943-024-01984-2

**Published:** 2024-05-08

**Authors:** Lisa-Marie Rieckmann, Michael Spohn, Lisa Ruff, David Agorku, Lisa Becker, Alina Borchers, Jenny Krause, Roisin O’Reilly, Jurek Hille, Janna-Lisa Velthaus-Rusik, Niklas Beumer, Armin Günther, Lena Willnow, Charles D. Imbusch, Peter Iglauer, Ronald Simon, Sören Franzenburg, Hauke Winter, Michael Thomas, Carsten Bokemeyer, Nicola Gagliani, Christian F. Krebs, Martin Sprick, Olaf Hardt, Sabine Riethdorf, Andreas Trumpp, Nikolas H. Stoecklein, Sven Peine, Philipp Rosenstiel, Klaus Pantel, Sonja Loges, Melanie Janning

**Affiliations:** 1 Department of Personalized Oncology, DKFZ-Hector Cancer Institute, University Medical Center Mannheim, Medical Faculty Mannheim, University of Heidelberg, Mannheim, Germany; 2 Division of Personalized Medical Oncology (A420), German Cancer Research Center (DKFZ), Heidelberg, Germany; 3 German Center for Lung Research (DZL), Heidelberg, Germany; 4 Department of Personalized Oncology, University Hospital Mannheim, Medical Faculty Mannheim, University of Heidelberg, Mannheim, Germany; 5 Bioinformatics Core, University Medical Center Hamburg-Eppendorf, Hamburg, Germany; 6 Clinic of Pediatric Hematology and Oncology, University Medical Center Hamburg-Eppendorf, Hamburg, Germany; 7 Research Institute Children’s Cancer Center Hamburg, Hamburg, Germany; 8 Department of Oncology, Hematology and Bone Marrow Transplantation With Section Pneumology, University Medical Center Hamburg-Eppendorf, Hamburg, Germany; 9 Miltenyi Biotec B.V. & Co. KG, R&D, Bergisch Gladbach, Germany; 10 Heidelberg Institute for Stem Cell Technology and Experimental Medicine (HI-STEM GmbH), Heidelberg, Germany; 11 Division of Stem Cells and Cancer, German Cancer Research Center, (DKFZ-ZMBH Alliance), Heidelberg, Germany; 12 III. Department of Medicine, University Medical Center Hamburg-Eppendorf, Hamburg, Germany; 13 Hamburg Center for Translational Immunology (HCTI), University Medical Center Hamburg-Eppendorf, Hamburg, Germany; 14 I. Department of Medicine, University Medical Center Hamburg-Eppendorf, Hamburg, Germany; 15 Department of Tumor Biology, University Medical Center Hamburg-Eppendorf, Hamburg, Germany; 16 Division of Applied Bioinformatics, German Cancer Research Center (DKFZ), Heidelberg, Germany; 17 Faculty of Biosciences, Heidelberg University, Heidelberg, Germany; 18 Institute of Pathology, University Medical Center Hamburg-Eppendorf, Hamburg, Germany; 19 Institute of Clinical Molecular Biology, Christian-Albrechts-University and University Hospital Schleswig-Holstein, Kiel, Germany; 20 Translational Lung Research Center Heidelberg, Member of the German Center for Lung Research (DZL), Department of Thoracic Oncology, Thoraxklinik at University Hospital Heidelberg, Heidelberg, Germany; 21 Department of General, Visceral and Thoracic Surgery, University Medical Center Hamburg-Eppendorf, Hamburg, Germany; 22 General, Visceral and Pediatric Surgery, University Hospital and Medical Faculty, Heinrich-Heine-University Düsseldorf, Düsseldorf, Germany; 23 Institute of Transfusion Medicine, University Medical Center Hamburg-Eppendorf, Hamburg, Germany

**Keywords:** Circulating tumor cells, Non-small cell lung cancer, Single cell RNA sequencing, Intratumor heterogeneity

## Abstract

**Background:**

Circulating tumor cells (CTCs) hold immense promise for unraveling tumor heterogeneity and understanding treatment resistance. However, conventional methods, especially in cancers like non-small cell lung cancer (NSCLC), often yield low CTC numbers, hindering comprehensive analyses. This study addresses this limitation by employing diagnostic leukapheresis (DLA) to cancer patients, enabling the screening of larger blood volumes. To leverage DLA’s full potential, this study introduces a novel approach for CTC enrichment from DLAs.

**Methods:**

DLA was applied to six advanced stage NSCLC patients. For an unbiased CTC enrichment, a two-step approach based on negative depletion of hematopoietic cells was used. Single-cell (sc) whole-transcriptome sequencing was performed, and CTCs were identified based on gene signatures and inferred copy number variations.

**Results:**

Remarkably, this innovative approach led to the identification of unprecedented 3,363 CTC transcriptomes. The extensive heterogeneity among CTCs was unveiled, highlighting distinct phenotypes related to the epithelial-mesenchymal transition (EMT) axis, stemness, immune responsiveness, and metabolism. Comparison with sc transcriptomes from primary NSCLC cells revealed that CTCs encapsulate the heterogeneity of their primary counterparts while maintaining unique CTC-specific phenotypes.

**Conclusions:**

In conclusion, this study pioneers a transformative method for enriching CTCs from DLA, resulting in a substantial increase in CTC numbers. This allowed the creation of the first-ever single-cell whole transcriptome in-depth characterization of the heterogeneity of over 3,300 NSCLC-CTCs. The findings not only confirm the diagnostic value of CTCs in monitoring tumor heterogeneity but also propose a CTC-specific signature that can be exploited for targeted CTC-directed therapies in the future. This comprehensive approach signifies a major leap forward, positioning CTCs as a key player in advancing our understanding of cancer dynamics and paving the way for tailored therapeutic interventions.

**Supplementary Information:**

The online version contains supplementary material available at 10.1186/s12943-024-01984-2.

## Background

Targeted therapy and immune checkpoint inhibitors have achieved remarkable success in treating non-small cell lung cancer (NSCLC); however, lung cancer remains the leading cause of cancer-related mortality globally [1]. While substantial progress has been made in the understanding of genomic-driven resistance through the broader use of next generation sequencing approaches, there is a critical unmet need to elucidate non-genomic-driven resistance mechanisms [2]. This endeavor has been hampered by the accessibility of appropriate tumor material, as the relapsed or progressed tumor sites are often inaccessible without significant biopsy-related risks. Additionally, single site biopsies inadequately represent patient’s intratumor heterogeneity. Accurate comprehension of tumor resistance mechanisms and underlying tumor heterogeneity is essential for developing novel effective therapies, given that tumor heterogeneity is a key driver of therapy resistance, leading to relapse and ultimately to patient death [3].

Circulating tumor cells (CTCs) hold great potential for addressing this challenge, as they can originate from primary tumors and metastases, offering a non-invasive access via the bloodstream [4]. However, current often epithelial cell adhesion molecule (EpCAM)-based detection methods, commonly used on peripheral blood (PB) samples, are limited to a few cancers with higher CTC numbers such as breast and prostate cancer, or very rare cases of patients with exceptionally high CTC numbers. EpCAM’s low expression on NSCLC-CTCs hampers its effectiveness as a marker for enriching CTCs, hindering the application of in-depth single-cell (sc) sequencing techniques crucial for monitoring tumor heterogeneity [4].

Recent reports suggest that diagnostic leukapheresis (DLA) holds potential for increasing CTC numbers [5]. The underlying assumption is that CTCs share density and cell size similarities with mononucleated cells (MNCs), making their collection feasible via leukapheresis [6]. However, the full potential of DLA has been limited thus far, as there is a scarcity of enrichment methods for CTCs from larger blood volumes. Standard enrichment techniques such as FDA-cleared CellSearch^®^ and Parsortix^®^ systems were designed for isolating CTCs from smaller PB samples (7.5- 9 ml PB).

In this study, we present a method for CTC enrichment from a mean of 20 × 10^8^ white blood cells (WBCs) obtained from DLAs (representing a 20-fold increase in WBCs compared to a typical PB sample), leading to an unprecedented number of 3363 sc CTC transcriptomes from six NSCLC patients. Thus, allowing for an in-depth characterization of CTC cell states, including cancer stemness and different metabolic types. We show that our novel DLA-pipeline enables non-invasive longitudinal analyses of CTCs and that it has the potential to transform personalized medicine for patients with metastatic NSCLC and beyond.

## Methods

A detailed overview of Materials and Methods can be found in the Supplementary section (Supplementary Material and Methods).

## Results

### Patient characteristics and enrichment of CTCs from diagnostic leukapheresis

DLA was performed on four NSCLC patients with adenocarcinoma and two NSCLC patients with squamous cell histology. At the time of the DLA, three of those patients had active disease (initial diagnosis or progression from previous treatment), while tumors from the remaining patients were controlled (Supplementary Table 1).

A mean blood volume of 6.0 L per patient was screened during DLA and a mean number of 64.6 × 10^8^ WBCs were collected. MNCs were sufficiently collected via DLA (mean MNC collection efficiency was 41% (range: 26 - 68%, Supplementary Table 2)). As described before, DLAs were generally well tolerated [6]. Blood samples taken before and after DLA indicated minor decreases in peripheral differential blood counts, but all numbers were within the normal range (Supplementary Fig. 1).

**Fig. 1:**
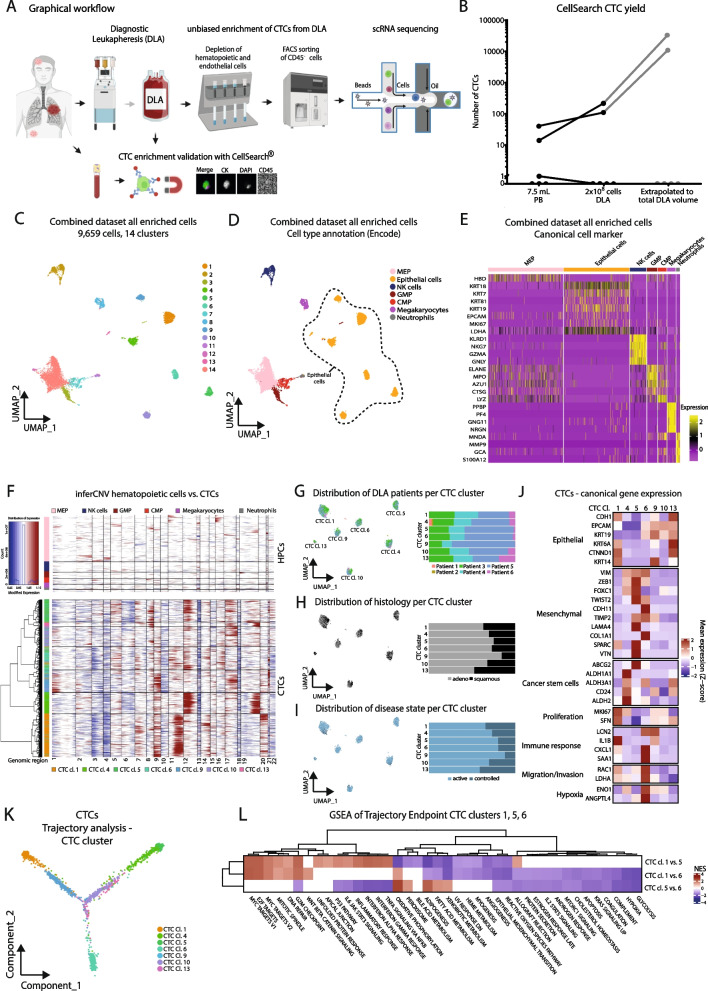
Identification and characterization of circulating tumor cells from NSCLC DLA samples. **A** Graphical overview of the CTC enrichment pipeline from DLAs. **B** Absolute numbers of CTCs detected by CellSearch^®^ from 7.5 ml of peripheral blood samples, taken prior to DLA procedures and 2 × 10^8^ cells, as well as the extrapolated number of CTCs to be expected in the total DLA product (in grey). Lines connect data from the same patient. **C** UMAP plot of all cells (*n* = 9,659) enriched from 6 DLAs from advanced stage NSCLC patients. Color-coding indicates the 14 different clusters. **D** UMAP plot of all cells colored by their cell types annotated by SingleR analysis (ENCODE reference). Dotted line encircles annotated epithelial cell /CTC clusters. **E** Heatmap of canonical cell marker. Yellow indicates high expression of a particular gene and purple indicates low expression. **F** inferCNV analysis [8] of CTCs and reference hematopoietic cells (MEPs, NK cells, GMPs, CMPs, neutrophils and platelets/megakaryocytes). **G-I** UMAP plot and bar charts of only the CTCs from all DLA samples colored by patient distribution (**G**) histology (**H**) and disease state (**I**). Active disease state active includes DLA performed at initial diagnosis and progression; controlled disease stage includes DLAs at timepoint characterized by stable disease or partial remission. **J** Heatmap of mean gene expression of canonical genes (epithelial, mesenchymal, cancer stem cell, proliferation, immune response, migration/invasion and hypoxia). **K** Trajectory analysis of CTCs. Color coding indicates the seven different CTC cluster along the branches **L** Gene set enrichment analysis using the HALLMARK gene set of DEG comparisons between trajectory endpoint clusters (cluster 1, 5 and 6 between each other) (red: high normalized enrichment score, blue: low normalized enrichment score). *MEP* Megakaryocyte/erythroid progenitor, *NK* natural killer, *GMP* granulocyte-monocyte progenitor, *CMP* common myeloid progenitor

For comparison, we also investigated CTCs from a 7.5 ml PB sample taken immediately prior to DLA and from 2 × 10^8^ cells of each DLA, using the “goldstandard”, FDA-cleared CellSearch^®^ system. In our cohort of advanced stage NSCLC patients, CTCs were detected in 2 out of 6 patients (33%), which is consistent with other studies using the EpCAM-based CellSearch^®^ system for detection of CTCs in patients with advanced stage NSCLC [7]. Using DLA increased CTC numbers in these patients (mean CTCs PB 9 (range 0–41) vs. mean CTCs DLA 54 (range 0–216) (Fig. 1B).

Because many NSCLC-CTCs lack expression of epithelial markers such as EpCAM [4], we purposely chose an unbiased approach based on a negative selection of CTCs by targeting hematopoietic cells (HPCs) for CTC enrichment from DLAs prior to sc RNA sequencing (scRNAseq) (Fig. 1A). First leukocytes (CD45^+^), T-cells (CD3^+^), endothelial cells (CD31^+^), monocytes/neutrophils (CD16^+^) and erythrocytes (CD235a^+^) were depleted using magnetic beads. Next, the remaining fraction was FACS sorted for live (DAPI^−^) and CD45^−^ cells. This population was then subjected to whole transcriptome scRNAseq via 10X Genomics technology.

### Identification of CTC whole transcriptomes

For identification of CTCs, we pooled sc transcriptomes from all six patients. Shared nearest neighbor modularity clustering revealed 14 distinct clusters containing a total of 9,659 cells (Fig. 1C). High cluster stability and consistency, especially of the CTC cluster, was confirmed by Jaccard Index analysis and Silhouette width (Supplementary Fig. 2A and B, Supplementary Table 3). We utilized reference-based cell type annotation comparing to a reference data set with 'SingleR' and canonical marker gene expression and identified 7 distinct epithelial cell clusters; other identified cell types included megakaryocytes, neutrophils, natural killer (NK) cells, megakaryocyte/erythroid progenitors (MEPs), common myeloid progenitors (CMPs)/pro-myelocytes (CMPs) and granulocyte-monocyte progenitors (GMPs) (Fig. 1D & E, Supplementary Fig. 3A & B, Supplementary Table 4). Trajectory analysis, which enables the study of dynamic changes in gene expression, revealed a separate branch of epithelial cells compared to HPC, confirming a different origin for these cells (Supplementary Fig. 3C & D). Additionally, comparison of differentially expressed genes (DEGs) between HPCs and CTCs indicated substantially higher levels of epithelial markers, including keratins as well as increased expression of cell cycle (*CCND1*) and proliferation-related genes (*SFN*) in epithelial cells. *S100A2, NQO1*, and *ID1* were also amongst the top DEGs. These genes are known to be expressed in epithelial cells, including respiratory cells and are associated with cancer progression [9, 10]. In contrast, HPCs exhibited higher expression of genes associated with hematopoietic lineages, such as *MPO* (myeloperoxidase), *DEFA3* (neutrophil defensin 3), and *HBA1* and *HBA2 (*hemoglobin subunits alpha 1 and 2, Supplementary Fig. 3E, Supplementary Table 5). We specifically also investigated endothelial and fibroblast maker genes, as these cells can be found in the peripheral blood. None of the marker genes were identified in either HPC clusters or CTC clusters, with the exception of *COL1A1*, which was partially expressed in CTC cluster 6 (Supplementary Fig. 3F). Since cells in CTC cluster 6 also expressed *EPCAM* and *KRT19*, these cells are of epithelial origin (Fig. 1J). Comparing inferred copy number variations (CNVs) from epithelial cells to reference HPCs revealed notable evidence for CNVs in epithelial cells. Thus, these cells are henceforth referred to as CTCs (Fig. 1F). Overall, a total number of 3,363 NSCLC-CTCs was identified. As a note, inferCNV analyses from CTCs with healthy lung epithelial cells as reference confirmed increased CNV in CTCs (Supplementary Fig. 3G). 

Integrating a publicly available healthy donor PBMC scRNA dataset into the DLA scRNAseq dataset, demonstrated that the seven previously identified distinct CTC clusters prevailed and exclusively consisted of cells from DLA patients, while HPCs from the DLA scRNAseq dataset formed clusters with HPCs from the healthy PBMC dataset (Supplementary Fig. 4). This further corroborates that our pipeline can be used for the specific identification of CTCs.

Notably, the Human Primary Cell Atlas (HPCA) reference within SingleR does not classify megakaryocytes per se, but platelets (Supplementary Fig. 3A). Since most platelets were excluded during enrichment, we tested whether cluster 8 (Fig. 1C) was composed of platelet-coated CTCs. The DEGs between cluster 8 (‘platelets’) and HPCs did not indicate any expression of epithelial marker genes such as keratins or *EPCAM*, thus contradicting this hypothesis (Supplementary Table 6). Hence, cluster 8 was classified as megakaryocytes.

### Characterization of CTCs reveals distinct phenotypes

Surprisingly, CTCs clustered independently of individual patient, primary tumor histology or disease state (Fig. 1G-I), indicating a cellular state-based CTC-intrinsic clustering. To further analyze the different CTC clusters, we performed in-depth characterization of their transcriptomic profiles by analyzing mean gene expression in combination with pathway- and trajectory analyses (Fig. 1J-L, Supplementary Fig. 3H & I, Supplementary Tables 7–10). A heatmap showing the top 10 positive marker genes of each CTC cluster revealed that genes associated with epithelial phenotypes (*KRT5, KRT6A*), stemness (*ALDH1A1*) and extracellular matrix remodeling (*VTN, MMP7, FBLN1*) were distinctive of different CTC clusters (Supplementary Fig. 3I, Supplementary Table 7).

This was further specified by comparison of canonical marker genes demonstrating varied expression among CTC clusters (Fig. 1J). While each cluster showed some expression of different epithelial genes, mesenchymal markers were predominantly expressed in CTC clusters 4, 5 and 6, with CTC cluster 4 exhibiting a cancer stem cell (CSC)-like phenotype and cluster 6 displaying markers of migration and hypoxia. CTC cluster 1 showed immune response marker upregulation and marker indicating increased proliferation. CTC cluster 9, 10 and 13 exhibit diverse phenotypes. They showed enriched epithelial markers, but also some upregulation of makers associated with cancer stemness, proliferation and hypoxia.

To enhance understanding of these phenotypes and their relationships, trajectory analysis was performed, elucidating that CTC clusters 1, 5 and 6 represent the endpoints of three distinct branches (Fig. 1K, Supplementary Fig. 3H). Next, we utilized GSEA to understand the distinctions between the three branches and clusters (Fig. 1L, Supplementary Tables 8–10). In comparison to CTC clusters 5 and 6, CTC cluster 1 enriched fewer genes related to the EMT pathway. However, there was an upregulation of genes associated with mitotic spindles, DNA repair, and E2F targets, coupled with increased activity in cell adhesion via apical junctions in CTC cluster 1. Based on the greater expression of genes involved in the interferon-α/γ-response pathways, CTC cluster 1 could further be characterized as being more immune responsive than CTC cluster 5. The immune response pathways were also more highly expressed in CTC cluster 6 than in CTC cluster 5. GSEA confirmed the mesenchymal phenotype of CTC clusters 5 and 6. Remarkably, despite both CTC clusters exhibiting a mesenchymal-like phenotype in comparison to CTC clusters 1, 10, 9 and 13, they were located at the two ends of the trajectory analysis, indicating substantial heterogeneity of mesenchymal CTCs. According to the GSEA, CTC cluster 5 enriched genes involved in oxidative phosphorylation, adipogenesis and fatty acid metabolism, while CTC cluster 6 encompassed genes related to hypoxia, glycolysis and ROS pathways. Additionally, compared with CTC cluster 1 and 6 cells, CTC cluster 5 cells exhibited decreased expression of genes involved in inflammatory response, interferon response α and γ, indicating a rather immune-evasive phenotype (Fig. 1L).

Taken together, these data indicated a high degree of phenotypic heterogeneity and a variety of CTC phenotypes were revealed: (i) epithelial-like, immune responsive and highly proliferative (CTC cluster 1), (ii) mesenchymal, oxidative phosphorylation, and immune evasive (CTC cluster 5), (iii) mesenchymal, invasive, and glycolytic (CTC cluster 6), and (iv) cancer-stem cell like (CTC cluster 4).

### CTCs demonstrate heterogeneity similar to primary tumor cells, while concurrently manifesting CTC-specific phenotypes

**Fig. 2:**
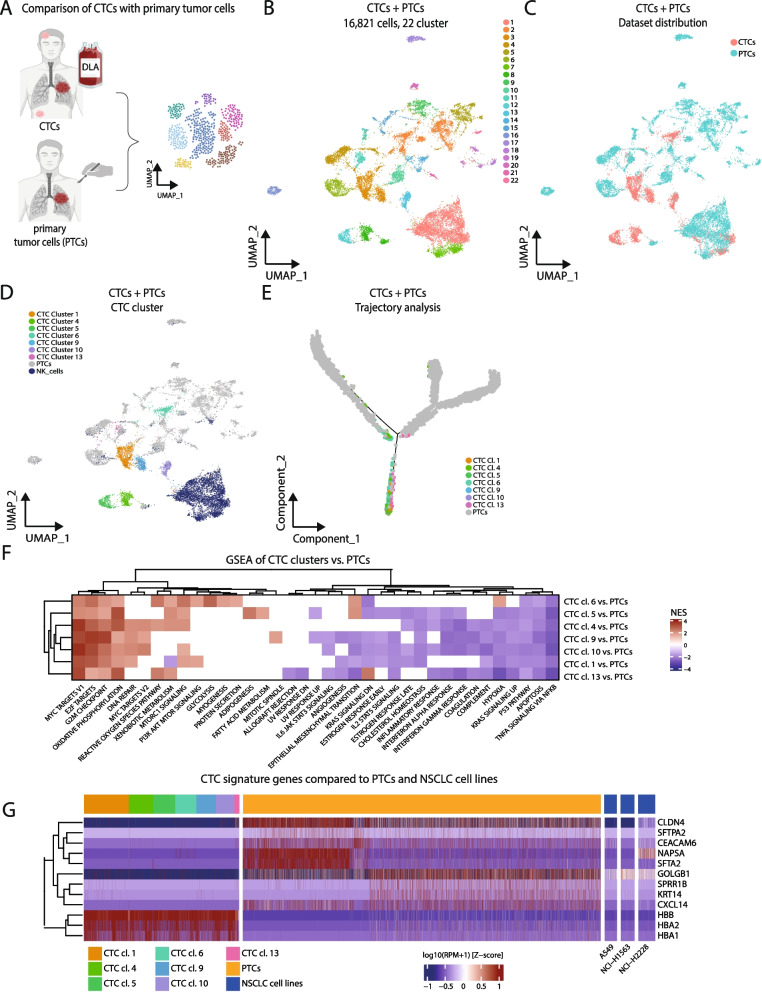
CTCs demonstrate heterogeneity similar to primary tumor cells, while concurrently manifesting CTC-specific phenotypes. ** A** Graphical overview: the primary tumor cell (PTC) scRNAseq dataset was comprised of scRNA data from *n* = 45 patients, including *n* = 42 from [12]. 5000 randomly selected PTCs were taken to match the size of the sc CTC-RNAseq dataset. The 'harmony' R package [11] was used for batch effect correction by incorporating NK cells (see also Supplementary Material and Methods for more information). **B** UMAP plot of scRNAseq data of CTCs from *n* = 6 DLAs and a subset of PTCs. **C** UMAP from B displaying the distribution of DLA CTCs (red) and PTCs (green). **D** UMAP from B colored by the distribution of NK cells (dark blue), PTCs (grey) and CTC clusters (rest of the colors). **E** Trajectory analysis of PTCs together with CTCs. Color coding indicates the seven different CTC cluster along the branches. **F** Unsupervised hierarchical clustering of GSEA analysis with the HALLMARK dataset based on DEGs of individual CTC cluster vs. all PTCs. (red: high normalized enrichment score, blue: low normalized enrichment score). **G** Heatmap showing the comparison of gene expression in CTC cluster, PTCs and NSCLC cell lines. Red indicates a high expression and blue indicates a low expression

To elucidate to which degree CTCs may exhibit distinct phenotypes in comparison to primary tumor cells (PTC), we analyzed the sc CTC-RNAseq dataset alongside an independent scRNAseq dataset from 45 primary NSCLC tumor samples (Supplementary Table 1). We randomly selected 5000 PTCs from the sc PTC-RNAseq dataset set to match the size of the sc CTC-RNAseq dataset, before the two datasets were integrated using the 'harmony' R package for batch effect correction by incorporating NK cells ([11] and Supplementary Material and Methods). This revealed 22 distinct cell clusters in an unsupervised clustering (Fig. 2A and B). As a note, Jaccard Index and Silhouette width indicated good cluster stability and consistency (Supplementary Fig. 2C & D, Supplementary Table 11).

Remarkably, this analysis indicated the preservation of most CTC clusters (Fig. 2B - D). CTC clusters 12 and 13 from this combined dataset comprised more than 90% CTCs, and CTC cluster 3 and 8 contained more than 75% of CTCs, reinforcing a distinctive phenotype of these CTC clusters (Supplementary Table 12). Notably, only CTC cluster 6 (mesenchymal, invasive and hypoxic) was integrated with PTCs (cluster 2 in Fig. 2B). A trajectory analysis further affirmed the separation of CTC clusters from PTCs, suggesting a potential distinctive phenotype for CTCs (Fig. 2E, Supplementary Fig. 5).

GSEA unveiled enriched pathways encompassing proliferation, translation (G2M checkpoint, DNA repair, E2F and Myc targets) and oxidative phosphorylation, while exhibiting decreased expression in TNFα signaling via NFκB, apoptosis and p53 pathways compared to PTCs (Fig. 2F, Supplementary Table 13). Noteworthy, immune-evasive phenotypes were observed in most CTC clusters, evidenced by reduced enrichment in inflammatory and interferon response pathways. Differential gene expression analysis highlighted genes related to hemoglobin metabolism, with *HBB* and its partners *HBA1* and *HBA2* highly expressed in CTCs, emphasizing a CTC-specific function (Fig. 2G). Conversely, genes involved in surfactant metabolism (*NAPSA, SFTPB, SFTPA2* and *SFTA2)* were downregulated in CTCs compared to PTCs. Interestingly, giantin (*GOLGB1*) which is associated with invasion and was shown to be higher expressed on breast cancer CTCs than on primary breast cancer cells, is also higher expressed in NSCLC-CTCs than primary NSCLC tumor cells (Fig. 2G, Supplementary Table 14).

Overall, these data corroborate the hypothesis that CTCs possess a distinct, immune-evasive phenotype, enabling them to adapt to the unique microenvironment and stresses in the bloodstream. On the other side, mesenchymal CTC clusters 5 and 6 exhibit increased expression of genes associated with EMT, while CTC clusters 9, 10 and 13 presented an overall lower expression of EMT genes compared to PTCs. CTC cluster 6, in particular, showcased a metabolically unique profile with elevated expression of anaerobic glycolysis genes despite oxygen availability in the bloodstream. Together, this also emphasizes the role of CTCs in representing heterogeneity of PTCs, particularly concerning EMT, metabolic disparities, and other relevant pathways.

## Discussion

Understanding of tumor heterogeneity and combating therapeutic resistance remain pivotal challenges in cancer treatment. Although CTCs hold great promise, their potential is hindered by their scarcity in standard peripheral blood samples, particularly in low CTC cancers like NSCLC [3, 4]. This is the first study representing a pioneering pipeline for analyzing larger DLA volumes, revealing the full potential of DLA and CTCs as pivotal diagnostic and translational tool in cancer research. Using this pipeline, we identified an unprecedented number of 3,363 CTCs from six NSCLC patients, by far the largest CTC dataset to date. In-depth sc transcriptomes revealed broad functional heterogeneity of CTCs along the EMT axis, including a cancer-stem cell like phenotype and different metabolic states. A comparison with PTCs indicated that CTCs indeed may represent intratumoral heterogeneity, while exhibiting specific CTC-related features.

Confirming prior studies, our findings underscored good tolerability of DLAs. In comparison to other studies based on identification of CTCs by epithelial markers [5, 6], our unbiased approach, focusing on depletion of HPCs and endothelial cells, led to the identification of large numbers of CTC, many of them with low or no *EPCAM* expression (Fig. 1J). The robustness of our analysis pipeline was confirmed by integration of scRNAseq data from healthy PBMCs with our negatively depleted DLA CTC dataset, which identified the same CTC clusters exclusively in the DLA dataset.

While previous studies showed patient-specific clustering in tissue tumor samples [12, 13], this study surprisingly reveals that NSCLC-CTC clustering is based on the specific tumor phenotype, not donor or clinical characteristics. This suggests that CTCs may be more dedifferentiated than primary tumors, supported by the loss of function of specific genes in CTCs, for instance genes involved in surfactant production (Fig. 2F and G, Supplementary Table 14).

Not surprisingly, CTC phenotypes represented cell states along the EMT continuum. However, only the exceptionally high number of total CTC transcriptomes, achieved through DLAs in combination with our unbiased enrichment, subsequently allowed for an in-depth and robust analysis of these cell states and representation of the full complexity of tumor heterogeneity, a prerequisite for fully understanding and monitor treatment escape and resistance mechanisms. The phenotypic plasticity of CTCs in relation to EMT, immune responsive and metabolic states as well as cancer stem cells also depicts the phenotypic changes that CTCs may endure throughout the metastatic cascade, as well as the plasticity necessary to survive treatment with anti-cancer agents and stress factors in the bloodstream [14].

Furthermore, comparison with PTCs revealed CTC specific phenotypes, such as a generally greater proliferation and translation but also an overall immune-evasive phenotype (lower expression of genes involved in TNFα signaling via NFκB and interferon-α/γ-response), which may be required for CTCs in order to avoid attack by immune cells in the peripheral blood. One such mechanism, the escape of CTCs from NK-cell surveillance by hijacking the HLA-E:CD94-NKG2a checkpoint was recently identified [15]. We further noted the strong upregulation of *HBB* in CTCs. Although surprising, this finding was described before and may indicate a potential strategy for CTCs to endure oxidative stress [16].

A limitation of this study is a potential underrepresentation of heterotypic CTC cluster, due to the depletion of HPCs as part of CTC cluster. CTC cluster are very rare events, but they may exert greater metastatic potential than single CTCs [17]. Furthermore, the absence of comparison with patient-matched normal (healthy) lung tissue and peripheral blood cells may underrepresent the individual heterogeneity. These questions will be addressed in future studies including longitudinal in-depth analyses of CTCs obtained from DLA.

To summarize, this study unravels distinct CTC phenotypes, illuminating a path towards potential future CTC-directed treatments. The observed heterogeneity in CTCs compared to PTCs underscores the robust potential of CTCs in diagnostic and translational research, which will enhance our understanding of metastatic NSCLC. Altogether, the here proposed DLA – CTC pipeline allows a real-time evaluation of patterns of response and resistance upon immunotherapy and/ or targeted therapies and has the potential to transform personalized medicine for metastatic NSCLC patients and beyond.

### Supplementary Information

**Supplementary Material 1**.

**Supplementary Material 2**.

**Supplementary Material 3**.

## Data Availability

The dataset supporting the conclusions of this article are included within the article’s additional files and are also available from the corresponding author on reasonable request.

## Abbreviations

BEAM: Branched expression analysis modeling
CMP: Common myeloid progenitor
BSA: Bovine serum albumin
CSC: Cancer stem cell
CTC: Circulating tumor cell
CNV: Copy number variation
DLA: Diagnostic leukapheresis
DEG: Differentially expressed gene
DMSO: Dimethyl sulfoxide
EDTA: Ethylenediaminetetraacetic acid
EMT: Epithelial–mesenchymal transition
FBS: Fetal bovine serum
FDR: False discovery rate
GSEA: Gene set enrichment analysis
GMP: Granulocyte-monocyte progenitor
HPC: Hematopoietic cells
HPCA: Human primary cell atlas
MEP: Megakaryocyte/erythroid progenitors
MNC: Mononucleated cell
NK: Natural killer cell
NSCLC: Non-small cell lung cancer
PB: Peripheral blood
PBS: Phosphate buffered saline
PBMC: Peripheral blood mononuclear cell
PTC: Primary tumor cell
PC: Principal component
sc: Single cell
WBC: White blood cell
