# Supplementary material for: Diagnostic leukapheresis reveals distinct phenotypes of NSCLC circulating tumor cells
Source: Mol Cancer. 2024 May 8;23:93. doi: 10.1186/s12943-024-01984-2 (PMC11077784; doi:10.1186/s12943-024-01984-2)
Supplement: Supplementary file 1 — Supplementary Material 1. [file 12943_2024_1984_MOESM1_ESM.pdf]

Supplementary Figure 1. Peripheral differential blood count of patients pre and post DLA

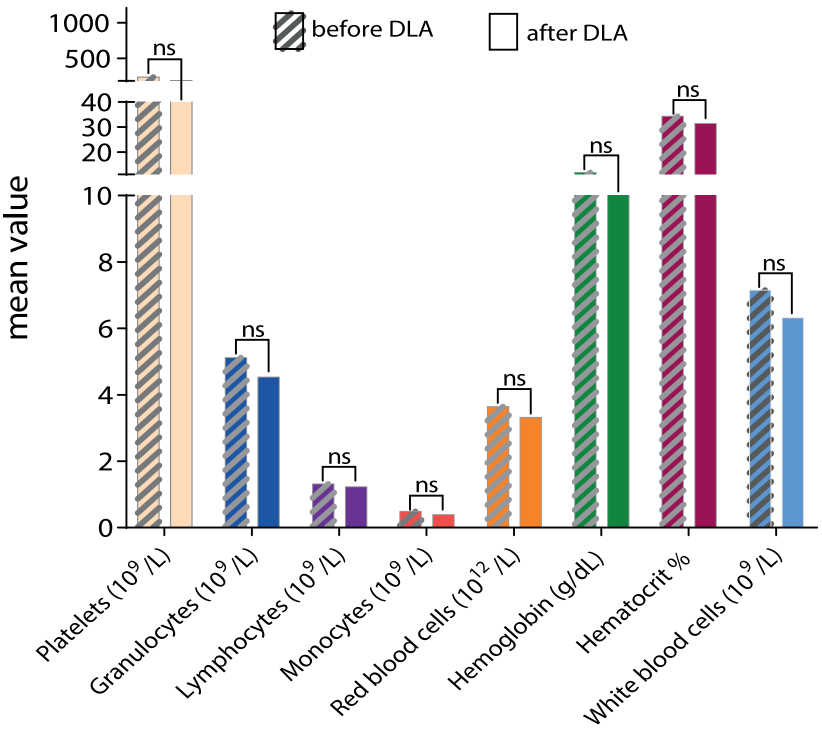

Bar chart indicating mean concentrations of peripheral blood cells from n=6 patients determined immediately pre and post DLA procedure.

Supplementary Figure 2. Cluster stability and consistency

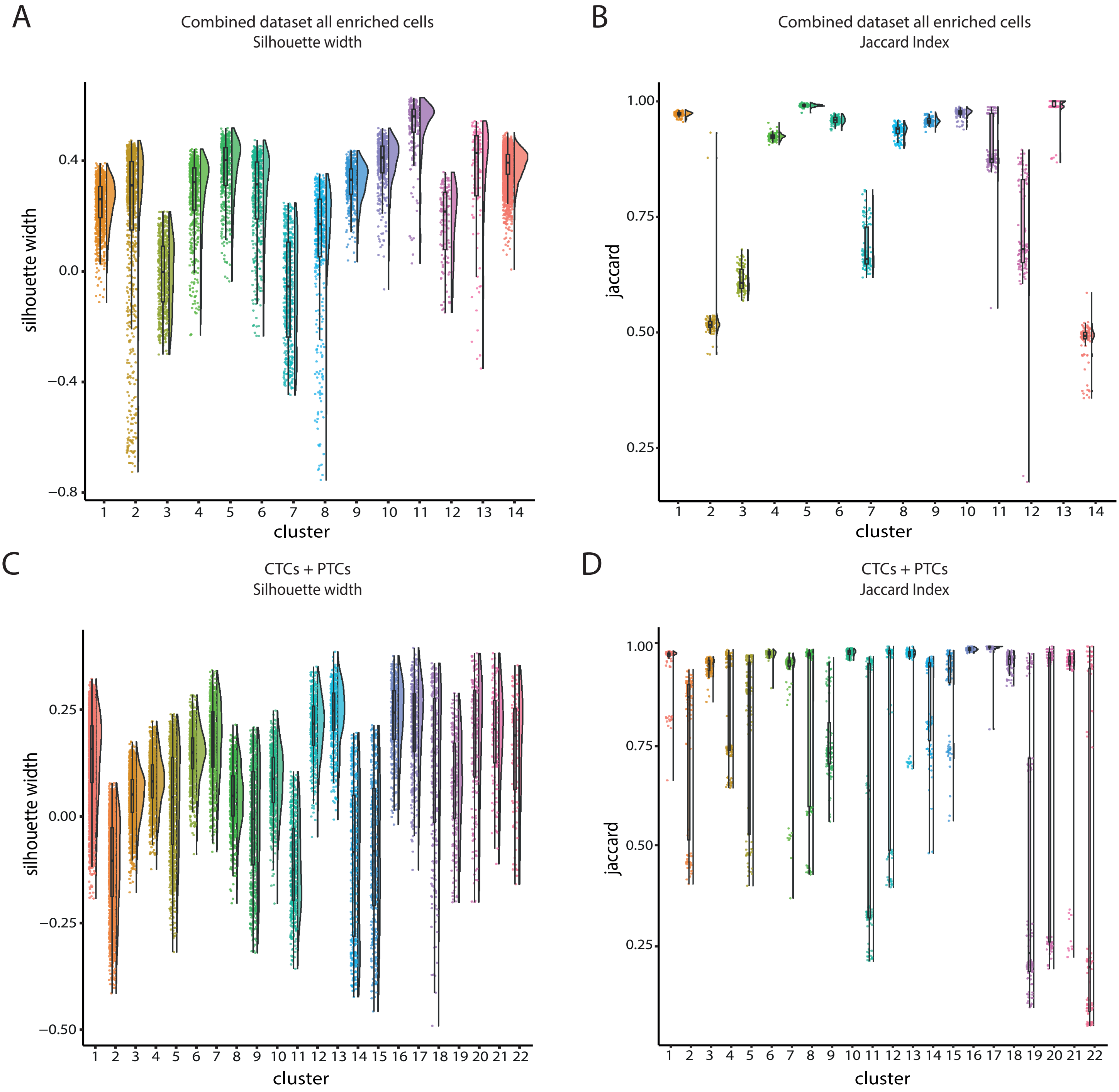

Tests to investigate cell cluster stability and consistency using (A) the silhouette width and (B) Jaccard index for the combined dataset with all enriched cells and in (C) and (D) for CTCs and PTCs, respectively.

Supplementary Figure 3. CTC definition, confirmation and characteristics

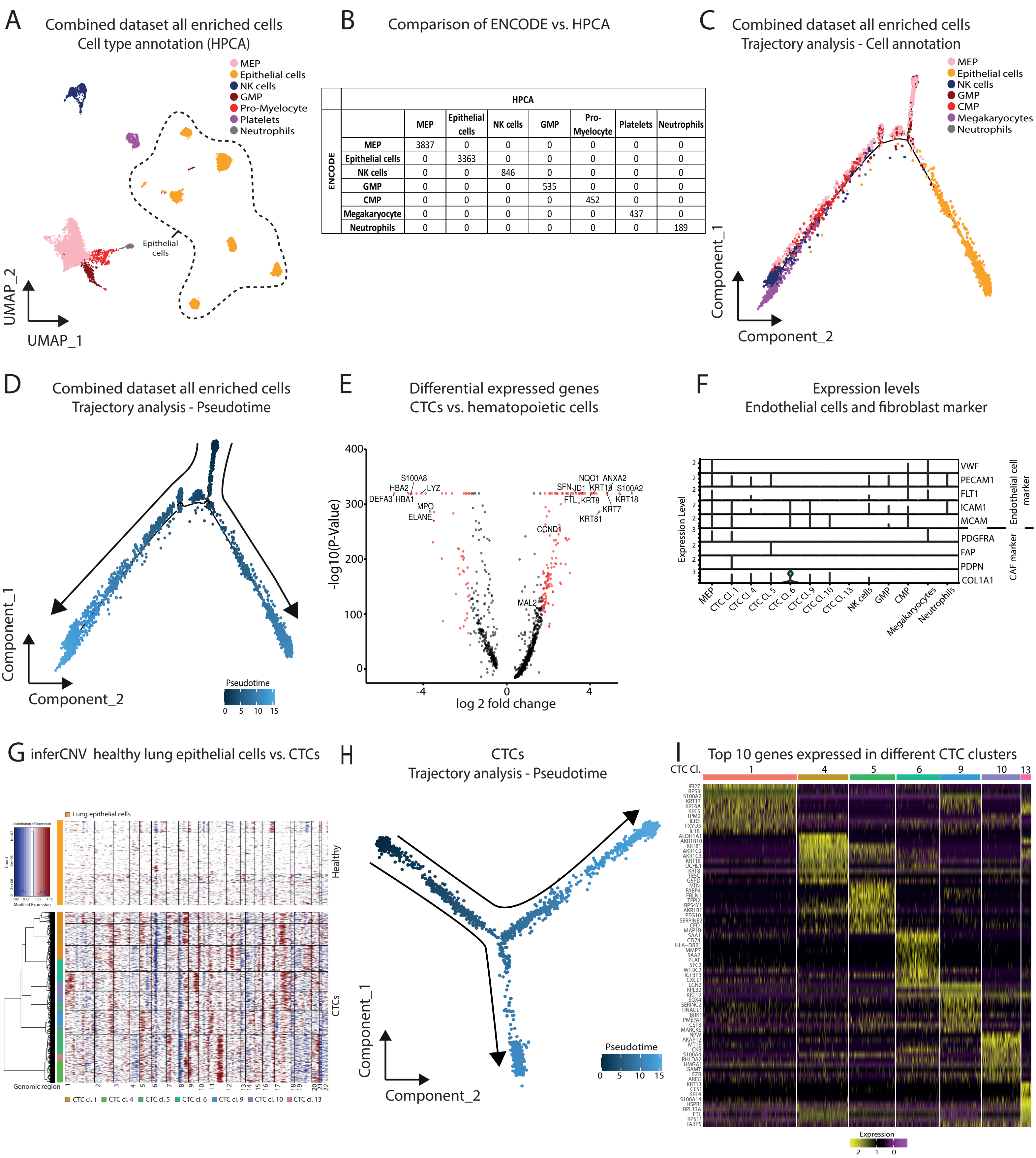

		HPCA						
		MEP	Epithelial cells	NK cells	GMP	Pro-Myelocyte	Platelets	Neutrophils
ENCODE	MEP	3837	0	0	0	0	0	0
	Epithelial cells	0	3363	0	0	0	0	0
	NK cells	0	0	846	0	0	0	0
	GMP	0	0	0	535	0	0	0
	CMP	0	0	0	0	452	0	0
	Megakaryocyte	0	0	0	0	0	437	0
	Neutrophils	0	0	0	0	0	0	189

Combined dataset of all enriched cells from DLA pipeline shown in (A) a UMAP color-coded by annotated cell type using SingleR with the Human Primary Cell Atlas reference dataset (dotted line indicates annotated epithelial cells), (B) direct cell number comparison of ENCODE vs. HPCA, trajectory analysis color-coded by (C) annotated cell type using SingleR with the Encode reference dataset and (D) pseudotime in a gradient with arrows underlining the biological evolution starting from progenitor cells (dark blue = start; light blue = end). (E) Differentially expressed genes comparing CTCs vs. hematopoietic cells. (F) Expression levels of endothelial and fibroblast marker in hematopoietic cells and single CTC cluster. (G) inferCNV analysis of CTCs using published healthy lung epithelial cells as a reference. (H) Trajectory analysis of only CTC clusters indicating the pseudotime colored in a gradient with arrows underlining the biological evolution starting from epithelial CTC cluster 1 (dark blue = start; light blue = end). (I) Top10 genes expressed in the different single CTC cluster.

Supplementary Figure 4. CTC identification algorithm in DLAs is specific to cancer patients

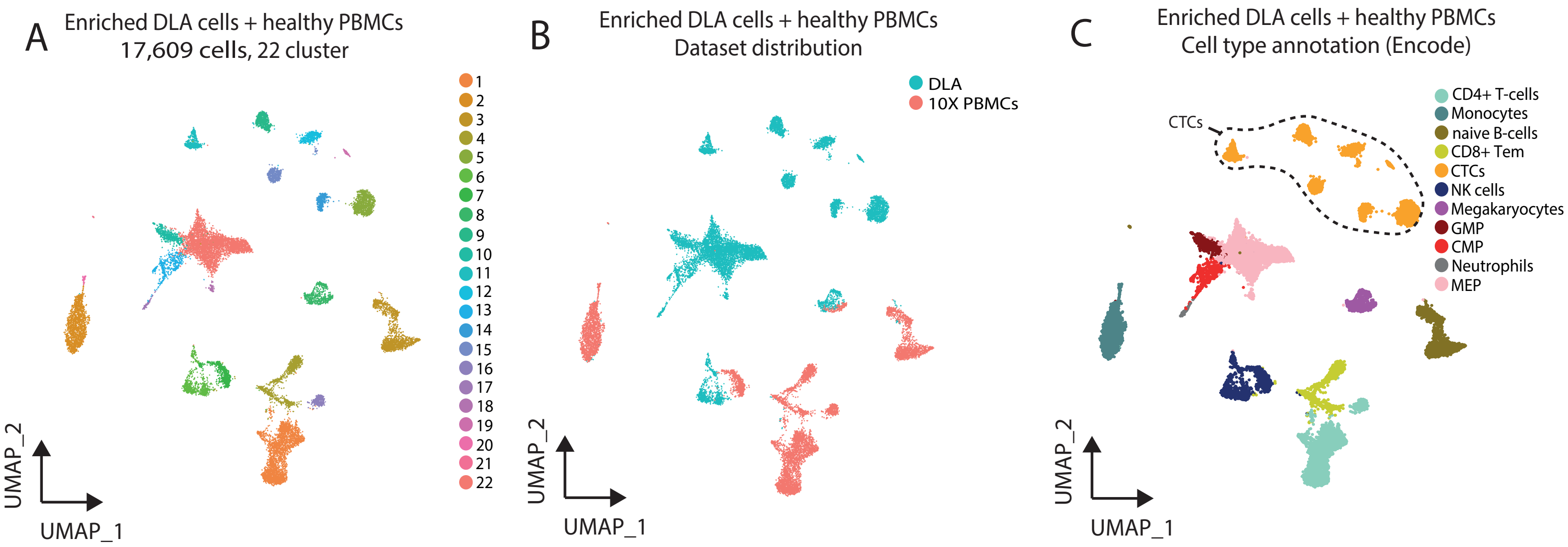

(A-C) UMAPs of enriched DLA cells together with publicly available healthy PBMCs color-coded by (A) clusters, (B) dataset distribution and (C) annotated cell type using SingleR with the ENCODE reference dataset. Dotted line identifies CTCs.

Supplementary Figure 5. Pseudotime of PTCs and CTCs

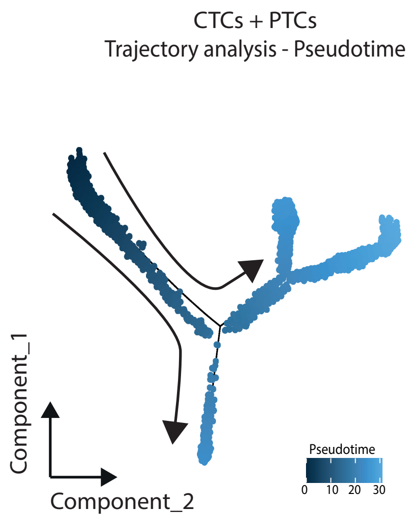

Trajectory analysis of CTCs and PTCs color-coded by pseudotime in a gradient with arrows underlining the biological evolution starting from primary tumor cells (dark blue = start; light blue = end).
