# Supplementary material for: Diagnostic leukapheresis reveals distinct phenotypes of NSCLC circulating tumor cells
Source: Mol Cancer. 2024 May 8;23:93. doi: 10.1186/s12943-024-01984-2 (PMC11077784; doi:10.1186/s12943-024-01984-2)
Supplement: Supplementary file 2 — Supplementary Material 2. [file 12943_2024_1984_MOESM2_ESM.docx]

**Supplementary Material and Methods**

**Cell culture**

Authenticity of NSCLC cell lines, NCI-H2228, NCI-H1563 and A549 was verified by SNP profiling at Multiplexion (Friedrichshafen, Germany). Cell lines were cultured under standard conditions in humidified incubators at 37°C with 5% CO_2_ and cultured with RPMI 1640 (Gibco, Life Technologies, Darmstadt, Germany) supplemented with 10% fetal bovine serum (Capricorn Scientific, Ebsdorfergrund, Germany). Cells were harvested using 0.25% trypsin-EDTA (Gibco, Life Technologies, Darmstadt, Germany) for 2-5 min at 37°C.

**DLA**

DLAs were performed on six stage IV NSCLC patients, all treated at the University Medical Center Hamburg between July 2019 and February 2020 (Supplementary Table 1). DLAs were performed at the Department of Transfusion Medicine of the University Medical Centre Hamburg-Eppendorf using the Spectra Optia® (Terumo BCT Inc., Lakewood, Colorado, USA) according to the manufacturer’s instructions. The apheresis machine was set to the same parameters as described previously [1] to enrich for white blood cells (WBCs) together with CTCs and to screen a patient’s total blood volume (TBV). Peripheral blood (PB) samples were taken before and after DLA to determine changes in differential blood counts.

**Detection of CTCs using CellSearch^®^**

Samples were analyzed as previously described [1, 2]. Briefly, PB samples were collected into CellSave™ tubes and subsequently analyzed for CTCs using the CTC kit according to the manufacturer’s instructions (Menarini Silicon Biosystems, Castel Maggiore, Italy). For DLA samples, volumes corresponding to 2 × 10^8^ cells of the DLA product, were transferred to CellSave™ tubes and diluted with CellSearch^®^ Circulating Tumor Cell Kit Dilution Buffer (both Menarini Silicon Biosystems, Castel Maggiore, Italy) up to a volume of 7.5 ml. The DLA samples were processed as “control” within the CellTracks^®^ AutoPrep^®^ System as instructed by the manufacturer.

**CTC enrichment from DLA for single-cell RNA sequencing**

A total of 80 × 10^8^ cells from each DLA were depleted from erythrocytes using diluted red blood cell lysis solution (Miltenyi Biotec, Bergisch Gladbach, Germany). For further depletion of hematopoietic cells (HPCs), the cells were incubated with magnetic microbeads targeting CD3 (130-050-101), CD16 (130-045-701), CD31 (130-091-935), CD45 (130-045-801) and CD235a (130-050-501) and subsequently processed on a MultiMACS Cell24 Separator Plus (all Miltenyi Biotec, Bergisch Gladbach, Germany). The depleted cell suspension was blocked in FACS buffer (2% fetal bovine serum (FBS), 0.09% NaN_3_, 0.82 mM ethylenediaminetetraacetic acid (EDTA) in phosphate-buffered saline (PBS), pH 7.4) supplemented with 10% human serum albumin (HSA; Jackson ImmunoResearch, St Thomas Place, United Kingdom) and stained with CD45-AlexaFluor647™ (clone HI30; BioLegend, San Diego, USA; 1:100) and EpCAM-AlexaFluor488™ (clone VU1D9, Cell Signaling Technology, Danvers, USA; 1:100) for 45 minutes at 4°C. To determine cell viability, the cells were also incubated with 4′,6-diamidino-2-phenylindole (DAPI, 1:100; Sigma-Aldrich, St. Louis, Missouri, USA). The cells were then sorted by fluorescence activated cell sorting on a FACSAria™ Fusion Cell Sorter (BD Biosciences, Franklin Lakes, New Jersey, USA) based on their CD45 negativity. This fraction was used immediately for downstream scRNAseq via 10X technology (10X Genomics, Pleasanton, California, USA).

**Dissociation of CT-guided needle biopsies from primary tumors**

Tumor biopsies from three NSCLC patients were collected at the Thoracic Clinic Heidelberg in March 2021 (Supplementary Table 1). Tissue biopsies were immediately transferred into CO_2_-independent medium (supplemented with 1% bovine serum albumin (BSA) and 1% glutamine, all from Gibco, Life Technologies, Darmstadt, Germany). The tissue was either dissociated using a combination of mechanical and enzymatic dissociation using the human Tumor Dissociation Kit in combination with the gentleMACS™ Octo Dissociator with heaters (both Miltenyi Biotec, Bergisch Gladbach, Germany) following the manufacturer’s instructions or by enzymatic only dissociation. For enzymatic only dissociation, biopsies were cut into pieces of 1-2 mm in size and incubated in an enzyme mixture (1X Hyaluronidase/Collagenase; Stemcell Technologies, Vancouver, Canada) supplemented with 0.015 mg/ml DNase I (Roche, Basel, Switzerland) in RPMI 1640 medium at 37°C for an hour with constant agitation. All cell suspensions were transferred to a new 50 ml tube after being passed through a 70 µm cell strainer. The single cell suspensions were washed with RPMI 1640 medium for removal of the enzymes, before being frozen in FBS/10% dimethylsulfoxid (DMSO). For scRNAseq, cell suspensions were quickly defrosted, washed and resuspended in PBS/0.4% BSA.

**10X Genomics: Chip Loading, cDNA and library prep; Sequencing**

Sorted single cell suspensions from DLA and thawed cell suspensions from tissue biopsies as well as NSCLC cells A549, NCI-H1563 and NCI-H2228 as controls were subjected to scRNA-seq using Chromium (10X Genomics, Pleasanton, California, USA) and Illumina NGS sequencing technologies (San Diego, California, USA). Single-cell suspensions were loaded onto a Chromium Single-cell Controller (10X Genomics, Pleasanton, California, USA) for the generation of single-cell gel beads in emulsions. Single-cell chip loading, gel bead in emulsion (GEM) generation and barcoding, post GEM-RT and cDNA amplification and library construction were performed according to the Chromium™ Single-cell 3′ Protocol - Chemistry v3. The constructed libraries were quantified using Qubit™ dsDNA High Sensitivity Assay Kit (Thermo Fisher Scientific, Waltham, Massachusetts, USA) and an Agilent DNA High Sensitivity Kit (Agilent, Santa Clara, California, USA). Next-generation sequencing was performed on an Illumina (San Diego, California, USA) NovaSeq6000 machine, yielding 2x100 bp paired-end reads.

**Single-cell RNA sequencing data analysis**

*Quality control*

After confirming the integrity of the raw reads by FastQC v0.11.8, they were mapped with CellRanger v3.0.2 to the human reference genome GRCh38 v3.0.0 provided by 10X Genomics. Further downstream processing was performed by R v3.6.0 and Bioconductor v3.10. First, the samples were read into the R environment with Seurat v3.2.0. Cells with fewer than 300 expressed genes, ≥ 15% unique molecular identifiers (UMI) expressed in mitochondrial genes and ≥ 10,000 UMIs were discarded. After all six samples were merged, 9659 cells were obtained with 23,353 expressed genes and 1337 UMIs per cell on average. Counts were normalized by the function “NormalizeData” and scaled by “ScaleData”.

*Unsupervised dimensionality reduction and clustering (UMAP)*

A total of 3,000 variably expressed genes were selected by the function “FindVariableFeatures” with default settings and subsequently used to compute the principal components (PCs). Based on “JackStraw” and “ElbowPlot” functions a subset of 50 significant PCs was selected and further used as dimensions of reduction when feasible. Cell clustering was performed using the “FindNeighbours” and “FindClusters” functions, with the resolution set to 0.3 for the latter one. To further ensure stable and consistent clustering, we used the chosen parameters to calculated Silhouette widths for each cell and Jaccard Index (n=100 with 80% of the cells of the full dataset) and employed the R-package scclusteval v1.0 for visualization [3]. For UMAP visualization the “RunUMAP” function was used.

*Cell type annotation*

For annotation, SingleR v1.0.6 was used with the built in Human Primary Cell Atlas (HPCA) and ENCODE as a reference data set [4, 5]). To annotate each cluster separately, the method was set to “cluster”, and the formerly obtained seurat clusters per cell were provided. For HPCA labels were chosen from the “label.main” slot and for ENCODE from the “label.fine” slot (Figure 1D, Supplementary Figure 3A and B).

*Single cell RNA sequencing data from primary tumor tissue and healthy lung epithelial cells*

For the comparison of CTCs with primary NSCLC tumor cells, we analyzed single-cell whole transcriptomes of cancer cells that had been defined based on canonical markers (EPCAM^+^, STNT^-^, CAPS^-^) from a published primary lung tumor dataset (n=42, [6]). Here, all the samples were obtained from stage IIIb/c and stage IV NSCLC patients, 83% of which were taken before any systemic treatment (Supplementary Table 1, [6]). Count matrices for each sample were obtained from GEO (GSE148071) and all datasets were processed as described above. We further included scRNA-Seq data from NSCLC tumor biopsies obtained from the Thoracic Clinic Heidelberg (n=3) (Supplementary Table 1). These data were processed in the same way as the primary tumor dataset from Wu et al. [6]. Initially, this dataset of primary tumor cells (PTCs) from 45 samples comprised transcriptomes of 56,421 cancer cells. For integration of the different scRNAseq datasets, we used the R package Harmony v0.1.0. NK cells from each dataset (total number= 4,546) were incorporated to confirm successful batch effect correction [7]. Cluster stability and consistency using Silhouette widths and Jaccard Index was used after batch correction.

Healthy lung epithelial cell data was extracted from Travaglini et al. [8]. In this work, normal lung tissues from uninvolved regions of freshly resected lung tissue from patients undergoing lobectomy for focal lung tumors were analyzed. The authors distinguished the epithelial cell population from stromal and immune cells by EpCAM expression. This dataset of healthy lung epithelial cells comprised transcriptomes of 9,407 cells.

*Single cell RNA sequencing data from publicly available healthy donor data*

We downloaded the count and feature matrices from a publicly available dataset with 10,000 cells from a healthy donor from the 10X Genomics website, which was processed with the same chemistry as the DLAs (10k PBMCs from a healthy donor (v3 chemistry)). The data were processed in the same way as the DLA datasets and was subsequently merged with the CTC data via the Seurat “merge” function.

*Trajectory analysis*

We applied Monocle2 v2.14.0 to the single-cell transcriptome data to determine the differentiation into developmental relationships of different cell types/ CTC phenotypes [9]. First, DEGs between the clusters defined by Seurat were estimated by the “differentialGeneTest” function. Only genes with a qval < 0.01 were selected for ordering and were set via the “setOrderingFilter” function. Afterwards, dimension reduction of the dataset was performed by the “reduceDimension” function, and the method was set to “DDRTree”. Finally, the trajectories were calculated by the “orderCells” function. Additionally, to determine the genes responsible for branch development, we performed the branched expression analysis modeling (BEAM) statistical test.

*Differentially expressed gene analysis*

Differentially expressed gene (DEG) analysis was done by the “FindMarkers” function of Seurat. Genes were considered DEGs when the false discovery rate (FDR), estimated by the Benjamini–Hochberg (BH) method, was less than 0.1.

*Gene set enrichment analysis*

The package fgsea v1.14.0 was used to perform GSEA [10]. Ranked lists of the log2-fold changes, estimated by the DEG analyses, were applied as input. Gene sets were obtained from the msigdbr package v7.2.1, and only the C1 HALLMARK sets were used [10].

*Copy number variation analysis*

We inferred copy number variations (CNVs) by inferCNV v1.10.1 using single-cell transcriptomic profiles [11]. Nonmalignant hematopoietic cells (MEP, CMP, GMP, neutrophils, NK cells and platelets) were used as baselines to estimate the CNVs of malignant cells. Briefly, genes were sorted by their genomic location on each chromosome. We then used 101 genes as a sliding window to smooth the relative expression on each chromosome to remove gene-specific expression influences. Genes with a mean expression less than 0.1 across all cells were excluded, as recommended for 10X Genomics data.

**References:**

1. Andree KC, Mentink A, Zeune LL et al. Toward a real liquid biopsy in metastatic breast and prostate cancer: Diagnostic LeukApheresis increases CTC yields in a European prospective multicenter study (CTCTrap). Int J Cancer 2018; 143: 2584-2591.

2. Fehm TN, Meier-Stiegen F, Driemel C et al. Diagnostic leukapheresis for CTC analysis in breast cancer patients: CTC frequency, clinical experiences and recommendations for standardized reporting. Cytometry Part A 2018; 93: 1213-1219.

3. Tang M, Kaymaz Y, Logeman BL et al. Evaluating single-cell cluster stability using the Jaccard similarity index. Bioinformatics 2021; 37: 2212-2214.

4. Aran D, Looney AP, Liu L et al. Reference-based analysis of lung single-cell sequencing reveals a transitional profibrotic macrophage. Nat Immunol 2019; 20: 163-172.

5. Dunham I, Kundaje A, Aldred SF et al. An integrated encyclopedia of DNA elements in the human genome. Nature 2012; 489: 57-74.

6. Wu F, Fan J, He Y et al. Single-cell profiling of tumor heterogeneity and the microenvironment in advanced non-small cell lung cancer. Nat Commun 2021; 12: 2540.

7. Korsunsky I, Millard N, Fan J et al. Fast, sensitive and accurate integration of single-cell data with Harmony. Nat Methods 2019; 16: 1289-1296.

8. Travaglini KJ, Nabhan AN, Penland L et al. A molecular cell atlas of the human lung from single-cell RNA sequencing. Nature 2020; 587: 619-625.

9. Qiu X, Mao Q, Tang Y et al. Reversed graph embedding resolves complex single-cell trajectories. Nat Methods 2017; 14: 979-982.

10. Liberzon A, Birger C, Thorvaldsdottir H et al. The Molecular Signatures Database (MSigDB) hallmark gene set collection. Cell Syst 2015; 1: 417-425.

11. Tickle T, Tirosh I, Georgescu C et al. inferCNV of the Trinity CTAT Project. In Klarman Cell Observatory BIoMaH (ed). Cambridge, MA, USA: 2019.
